# Improvement of p-Type AlGaN Conductivity with an Alternating Mg-Doped/Un-Doped AlGaN Layer Structure

**DOI:** 10.3390/mi12070835

**Published:** 2021-07-18

**Authors:** Chi-Chung Chen, Yu-Ren Lin, Yu-Wei Lin, Yu-Cheng Su, Chung-Chi Chen, Ting-Chun Huang, Ping-Hsiu Wu, C. C. Yang, Shin Mou, Kent L. Averett

**Affiliations:** 1 Institute of Photonics and Optoelectronics, Department of Electrical Engineering, National Taiwan University, No. 1, Section 4, Roosevelt Road, Taipei 10617, Taiwan; r05941083@ntu.edu.tw (C.-C.C.); r06941057@ntu.edu.tw (Y.-R.L.); r06941042@ntu.edu.tw (Y.-W.L.); r06941094@ntu.edu.tw (Y.-C.S.); r07941087@ntu.edu.tw (C.-C.C.); r07941101@ntu.edu.tw (T.-C.H.); r07941097@ntu.edu.tw (P.-H.W.); 2 Air Force Research Laboratory, Materials and Manufacturing Directorate, Wright-Patterson Air Force Base, Dayton, OH 45433, USA; shin.mou.1@us.af.mil (S.M.); kent.averett@us.af.mil (K.L.A.)

**Keywords:** p-type AlGaN, Mg doping, alternating-layer structure, hole mobility, sheet resistance

## Abstract

Using molecular beam epitaxy, we prepared seven p-type AlGaN samples of ~25% in Al content, including six samples with Mg-doped/un-doped AlGaN alternating-layer structures of different layer-thickness combinations, for comparing their p-type performances. Lower sheet resistance and higher effective hole mobility are obtained in a layer-structured sample, when compared with the reference sample of uniform Mg doping. The improved p-type performance in a layer-structured sample is attributed to the diffusion of holes generated in an Mg-doped layer into the neighboring un-doped layers, in which hole mobility is significantly higher because of weak ionized impurity scattering. Among the layer-structured samples, that of 6/4 nm in Mg-doped/un-doped thickness results in the lowest sheet resistance (the highest effective hole mobility), which is 4.83 times lower (4.57 times higher) when compared with the sample of uniform doping. The effects of the Mg-doped/un-doped layer structure on p-type performance in AlGaN and GaN are compared.

## 1. Introduction

Ultraviolet (UV) light is useful for many applications, including water, air, and surface sterilizations, medical light therapy, forensic analysis, drug discovery, DNA sequencing, etc. The fabrication of solid-state source in the UV range below 365 nm requires the ternary compounds of AlGaN [1,2]. So far, the efficiencies of UV light-emitting diodes (LEDs) based on AlGaN are still quite low, particularly those in the deep-UV wavelength range (<300 nm) [3,4,5]. Among several crucial factors for such a low efficiency, the difficulty of growing high-conductivity Mg-doped p-AlGaN is an important issue. Due to this difficulty, usually a p-GaN layer is applied to the top of such a UV-LED for improving its electrical behavior or reducing its device resistance. However, this p-GaN layer can absorb the emitted light from an AlGaN quantum well and decrease the light extraction efficiency of the LED. Therefore, enhancing the conductivity of Mg-doped AlGaN is a crucial issue for improving the performance of an AlGaN-based UV-LED. This issue is particularly important as high-quality transparent conductor in the UV range is still rare.

Besides the conventional Mg-doping method for fabricating p-type AlGaN, an AlGaN structure with decreasing Al content along the c-axis has been proposed for producing polarization-induced holes [6,7,8,9,10]. This approach uses the gradient of internal polarization field for generating a continuous hole gas distribution. In growing an AlGaN layer with an Al-content gradient for generating a p-type behavior, this gradient (in %Al/nm) is a crucial factor. It has been shown that the p-type conductivity of such an AlGaN layer increased with increasing Al-content gradient. It was observed that for a gradient lower than 0.28% Al/nm, the native n-type donors could hinder the polarization induced p-type behavior [10]. Therefore, AlGaN crystal quality is important for achieving a polarization induced p-type behavior. In UV-LED application, the available Al-content range for forming a p-type AlGaN with an Al-content gradient is limited to avoid the absorption of emitted UV light. For instance, if the emission wavelength of an AlGaN quantum well is 260 nm, the Al content for forming a polarization-induced p-type AlGaN layer to integrate with the quantum well must be higher than ~57%, which corresponds to the AlGaN bandgap of 4.77 eV [11]. This limitation may make the usable Al content range small and hence the polarization-induced behavior weak. Therefore, Mg-doping for fabricating p-type AlGaN is still a technique deserving further development.

One of the major causes for the low conductivity of Mg-doped AlGaN is the increasing activation energy of Mg acceptor in AlGaN with increasing Al content from ~170 meV in GaN to ~510 meV in AlN [12,13]. The high activation energy leads to a relatively lower hole concentration even though Mg doping concentration is high and hence a low conductivity level according to the equation,

(1) $$\sigma =qn\mu \;\mathrm{or}\;R=\frac{1}{qN\mu }$$

here, *σ* is the conductivity, *q* is the electron charge, *n* is the hole concentration, *R* is the sheet resistance, *N* is the sheet hole concentration, and *μ* is the hole mobility. Following the trace of developing p-GaN [14,15,16,17,18,19,20], a few approaches have been developed for improving the p-type conductivity of Mg-doped AlGaN, such as the technique of delta-doping [15]. With metalorganic chemical vapor deposition (MOCVD), the resistivity of a high-Al AlGaN sample could be reduced by using a high V/III ratio and a moderate level of Mg doping [21]. Based on the method of indium-surfactant-assisted Mg delta-doping with MOCVD in an Al_0.4_Ga_0.6x_N sample, the hole concentration could reach 4.75 × 10^18^ cm^−3^ and the sheet resistance of 2.46 × 10^4^ Ω/sq was achieved [16]. Using molecular beam epitaxy (MBE), liquid-metal-enabled synthesis of 70-% Al content AlGaN could result in a hole concentration of 6 × 10^17^ cm^−3^ [22,23]. Also, with MBE growth for 60%-Al AlGaN, hole concentration up to 8.7 × 10^17^ cm^−3^, hole mobility in the range of 10–17 cm^2^/V-s, and a minimum resistivity of 0.7 Ω-cm were achieved [24]. Meanwhile, based on MOCVD growth, a metal-source flow-rate modulation epitaxy method was used for growing 43%-Al AlGaN to achieve a hole concentration of 2.3 × 10^17^ cm^−3^ and resistivity of 12.7 Ω-cm [25]. To improve Mg-doped p-type conductivity of AlGaN, past efforts were mainly focused on the increase in Mg doping concentration or the reduction of activation energy. However, usually the increase of Mg doping or the enhancement of activation efficiency, i.e., the increase of hole concentration, leads to stronger hole scattering by ionized and non-ionized impurities, resulting in the reduction of hole mobility [26,27]. According to Equation (1), while the increase of hole concentration can enhance the conductivity, the reduction of hole mobility leads to a lower conductivity level. Unless hole mobility can be maintained or enhanced, it is difficult to significantly improve the conductivity of Mg-doped p-AlGaN by simply increasing hole concentration.

Recently, a concept that separates the locations of effective hole generation and high hole mobility for obtaining high effective conductivity has been demonstrated [28]. By growing a nanometer-scale Mg-doped/un-doped GaN alternating-layer structure, holes generated in an Mg-doped GaN layer can diffuse into the neighboring un-doped GaN layers, in which hole mobility is significantly higher because of weak impurity scattering. By combining the effective hole generation in Mg-doped GaN layers and the high hole mobility in un-doped layers, record-low p-type GaN resistivity of 0.038 Ω-cm was achieved [28]. In the current research, we apply the same concept to the growth of Mg-doped p-type AlGaN for improving its conductivity. In Section 2 of this paper, the sample structures, growth conditions, and material characterization results are presented. The Hall measurement results are reported in Section 3. Further discussions about the results are made in Section 4. Finally, the conclusions are drawn in Section 5.

## 2. Sample Structures, Growth Conditions, and Material Characterization Results

**Figure 1:**
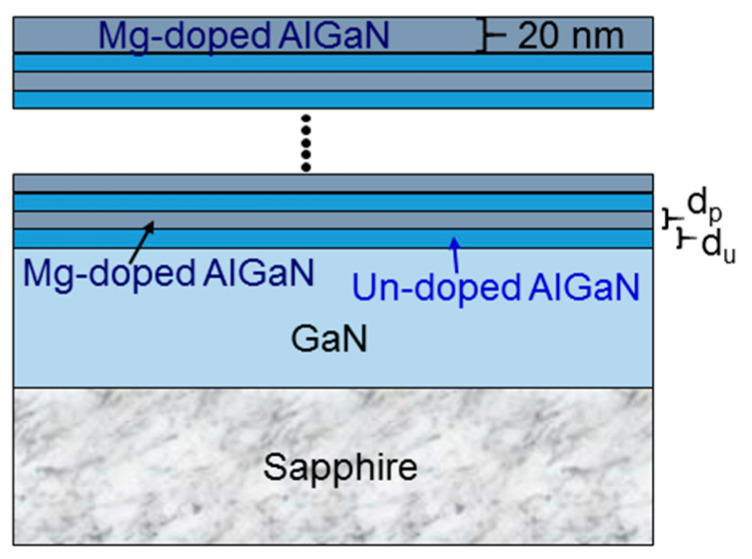
Schematic illustration of the structure of an Mg-doped/un-doped AlGaN layer-structured sample.

Eight AlGaN samples are prepared with MBE for comparison, including an un-doped AlGaN, a uniformly Mg-doped AlGaN, and six alternating Mg-doped/un-doped AlGaN layer-structured samples. All the samples are grown on GaN templates, which are fabricated with MOCVD on double-polished c-plane sapphire substrates. For preparing a GaN template, after a buffer layer growth at 530 °C on sapphire substrate, a GaN layer of ~4 micron in thickness is deposited at 1060 °C. As listed in row 1 of Table 1, the uniformly un-doped and uniformly Mg-doped AlGaN structures are designated as samples uf-u and uf-p, respectively. In either sample, the growth time for the 250-nm thickness is 120 min, corresponding to a growth rate of ~2.08 nm/min. As schematically illustrated in Figure 1, an alternating-layer structure, designated as sample d_p_/d_u_, consists of a certain period number of Mg-doped and un-doped AlGaN layers with d_p_ and d_u_ for the layer thicknesses, respectively, as also listed in row 1 of Table 1. For instance, the thickness of a single Mg-doped (un-doped) layer of sample 6/4 is 6 (4) nm. In each layer-structured sample, the MBE growth on a GaN template starts with an un-doped AlGaN layer of d_u_ in thickness and ends with an Mg-doped AlGaN layer of 20 nm in thickness. The growth period number is chosen to make the total AlGaN thickness close to 250 nm. The growth period numbers for the layer-structured samples are listed in row 2 of Table 1. Row 3 of Table 1 shows the total AlGaN thicknesses of all the samples under study. In MBE growth, the substrate temperature, *T*_sub_, is 745 (742) °C for growing an un-doped (Mg-doped) AlGaN layer. The Ga effusion cell temperature, *T*_Ga_, is 1048 (1062) °C for growing an un-doped (Mg-doped) AlGaN layer. For either un-doped or Mg-doped growth, the Al effusion cell temperature, *T*_Al_, is fixed at 935 °C. For Mg-doped growth, the Mg effusion cell temperature, *T*_Mg_, is fixed at 340 °C. The plasma power is 250 W and N_2_ flow rate is 0.6 sccm.

**Figure 2:**
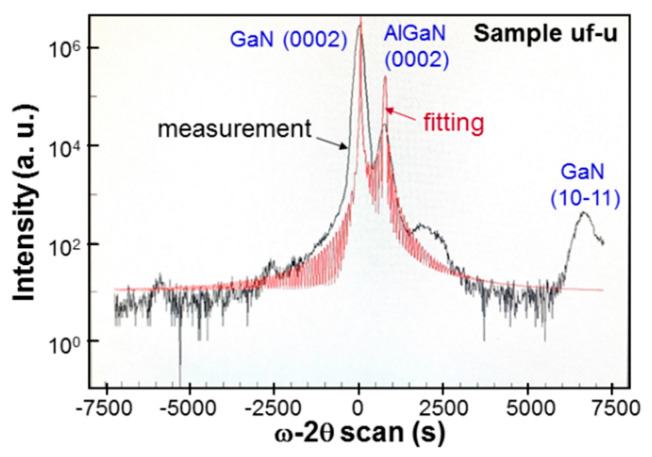
The results of ω-2θ scan in X-ray diffraction (XRD) measurement for sample uf-u. The black (red) curve shows the measurement data (fitting curve).

**Figure 3:**
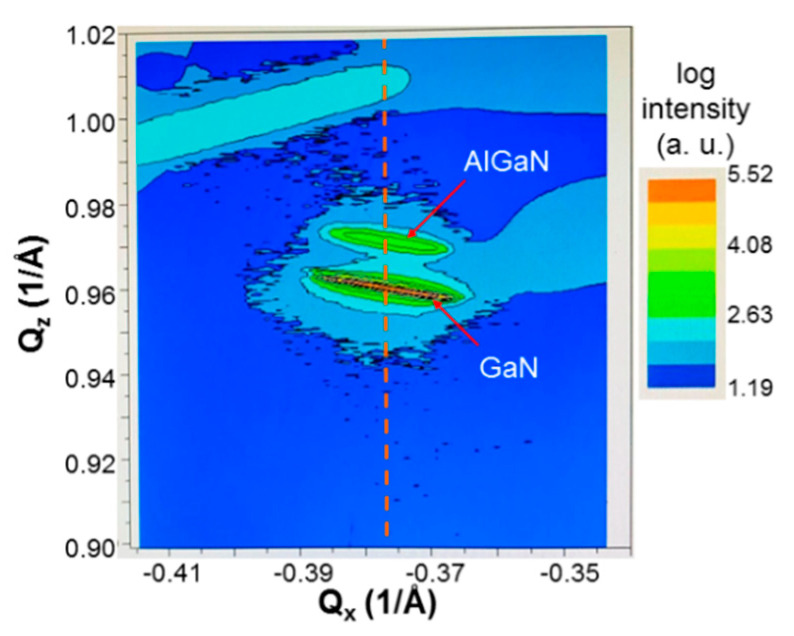
Reciprocal space mapping (RSM) result of sample uf-p. The vertical dashed line passing through the centers of the GaN and AlGaN features indicates that the AlGaN layer on the GaN template is fully strained.

The Al effusion cell temperature is adjusted for controlling the Al content of AlGaN at around 25%. Figure 2 shows the ω-2θ scan result in X-ray diffraction (XRD) measurement of sample uf-u. Here, the black and red curves correspond to the measurement and fitting results, respectively. The crystal planes for the scan features are labeled in the figure. The origins of the three un-labeled minor humps (two left to and one right to the main peaks) are unknown. The XRD peak of GaN 10–11 and another feature between 1250 and 2500 s (not labeled) in Figure 2 can be observed in the used GaN template. Therefore, they have nothing to do with AlGaN growth. It is speculated that such GaN crystal features were produced when the thick GaN layer was grown on sapphire substrate with MOCVD. In this process, before GaN growth reached a stable condition, other crystal structures might exist in the template, leading to the observations of these two features. From the fitting result of the AlGaN 0002 feature, we learn that the Al content of this sample is 24.7%. The ω-2θ scans of XRD measurement are performed for all other samples to give Al contents all around 25%. The XRD ω-scan results of all the samples are also obtained to give the full-widths at half-maximum (FWHMs) of the scan patterns in row 4 of Table 1. Here, one can see that after Mg doping, the ω-scan FWHM is slightly increased. In a layer-structured sample, the ω-scan FWHM is further increased. Either a thinner Mg-doped or un-doped layer in a layer-structured sample leads to a larger ω-scan FWHM. Figure 3 shows the reciprocal space mapping (RSM) result of sample uf-p. Here, the two features around the figure center correspond to the AlGaN and GaN crystals. The vertical dashed line connects the centers of the AlGaN and GaN features, indicating that the 250-nm ~25%-Al AlGaN grown on the GaN template is fully strained. Based on the similar RSM results of other samples, we find that the ~250 nm AlGaN with ~25% Al on a GaN template is always fully strained no matter it is un-doped, uniformly doped, or with modulated doping. In other words, the critical thickness of 25%-Al AlGaN grown on a GaN template is significantly larger than 250 nm.

**Figure 4:**
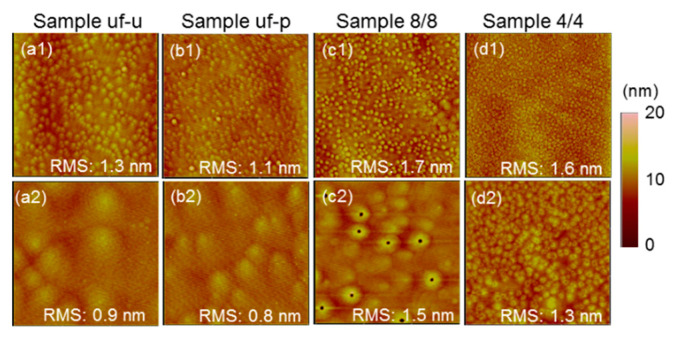
(**a1**–**d1**) (**a2**–**d2**) atomic force microscopy (AFM) images of samples uf-u, uf-p, 8/8, and 4/4, respectively, with (15 μm × 15 μm) (3 μm × 3 μm) in image dimension.

Figure 4(a1,a2) show the atomic force microscopy (AFM) images of sample uf-u with the dimensions of 15 μm × 15 μm and 3 μm × 3 μm, respectively. Figure 4(b1,b2), show the results similar to those in Figure 4(a1,a2), respectively, for sample uf-p. In Figure 4(c1,c2), and Figure 4(d1,d2), the similar results for sample 8/8 (4/4) are shown. A bright region in those AFM images corresponds to a hillock on the surface. It has been reported that around a threading dislocation, the AlGaN growth rate becomes higher [29]. Therefore, a threading dislocation may exist beneath a hillock. Generally, a threading dislocation ends on a sample surface with a V-pit. However, under our metal-rich growth condition, a V-pit can be filled up to show a hillock structure. In Figure 4(a1,a2,b1,b2), for sample uf-u (uf-p), only hillocks are observed. However, in Figure 4(c1,c2,d1,d2), for sample 8/8 (4/4), tiny dark spots around the centers of certain hillocks can be observed. Those dark spots correspond to V-pits. Actually, on the surfaces of all the layer-structured samples under study, V-pits are observed. In Figure 4(d1,d2), for sample 4/4, one can see that the dimension of high-density hillocks is significantly smaller than those in the other three samples of Figure 4. The hillock size of sample 4/4 is smaller than those of all other samples under study. V-pits can be observed in sample 4/4 even though they are quite small. In row 5 of Table 1, the numbers before and after slashes correspond to the root-mean-square roughness levels based on the AFM scans of 15 μm × 15 μm and 3 μm × 3 μm, respectively, in dimension. Generally, the surface roughness in a layer-structured sample is larger, when compared with sample uf-u or uf-p. In row 6 of Table 1, we show the surface V-pit densities of those layer-structured samples. Also, we show the surface hillock densities within the parentheses for samples uf-u and uf-p. The hillock or V-pit densities in all the samples under study are on the order of 10^8^ cm^−2^. One can see that with Mg doping in sample uf-p, the hillock density is increased, when compared with sample uf-u. Also, except sample 10/10, the V-pit density increases with decreasing layer thickness in the layer-structured samples.

**Figure 5:**
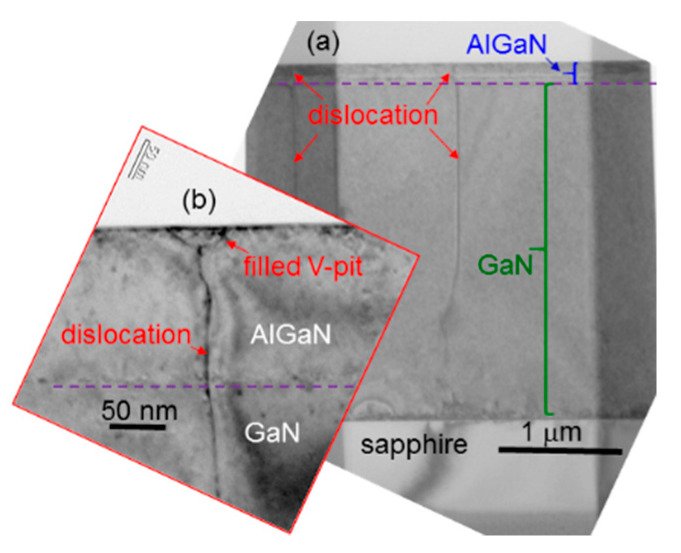
(**a**) Transmission electron microscopy (TEM) image showing that two dislocations extend from the GaN template into the Mg-doped AlGaN layer in sample uf-p. (**b**) Another TEM image of sample uf-p showing that a dislocation ends at the top surface with a filled V-pit.

**Figure 6:**
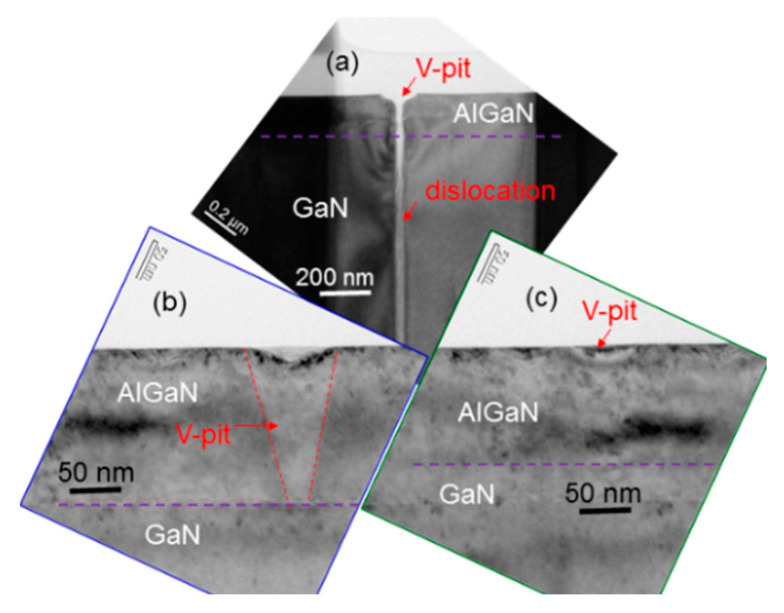
(**a**): TEM image of sample 8/8 showing the structure of a V-pit with a dislocation beneath. (**b**) and (**c**): TEM images of sample 8/8 showing the structures of a deep and a shallow V-pits, respectively, without dislocation beneath.

In Figure 5a, we show a cross-sectional transmission electron microscopy (TEM) image of sample uf-p, demonstrating the extension of two dislocations from the GaN template into the overgrown Mg-doped AlGaN layer. Here, the horizontal dashed line roughly divides the layers of AlGaN and GaN. In a few TEM images, we observe that dislocations in the Mg-doped AlGaN layer always originate from the GaN template. The TEM image in Figure 5b shows the structure of a dislocation around its top end. The V-shaped structure at the top end of the dislocation looks like a filled V-pit. This image may support the result of no V-pit observation in the AFM images of this sample shown in Figure 4(b1,b2). In this sample, most V-pits can be filled up to show hillock structures under the used metal-rich growth condition. Figure 6a shows a TEM image of sample 8/8 demonstrating an un-filled V-pit structure with a dislocation beneath. Then, in Figure 6b,c, we show the formation of a deep (shallow) V-pit in sample 8/8. The formation of the deep V-pit starts at the beginning of AlGaN growth. The shallow V-pit is formed near the top surface. However, no dislocation is observed beneath either deep or shallow V-pit. It is speculated that similar to a dislocation structure, the growth rate around a dislocation-free V-pit also becomes higher to consume the supplied constituent atoms of a fixed amount per surface area such that a hillock is formed around such a V-pit. Therefore, the growth of a layered structure can lead to the formation of a V-pit without a dislocation beneath. The formation of such a deep V-pit can be attributed to the lattice mismatch between the GaN template and the overgrown AlGaN. The formation of a shallow V-pit can be caused by the slight lattice variation between Mg-doped and un-doped layers. It is noted that we use the individually optimized growth conditions for depositing Mg-doped and un-doped layers. The different growth conditions lead to slightly different lattice structures between Mg-doped and un-doped layers. The accumulation of such small lattice variations can result in the formation of a shallow V-pit, as shown in Figure 6c. In this regard, a layered sample with a smaller layer thickness or a larger layer-period number can lead to a stronger accumulated lattice variation effect such that its V-pit density becomes higher. This speculation is roughly supported by the data in row 6 of Table 1, particularly those of samples 4/4, 6/6, and 8/8. However, the mechanism for such a behavior is still unclear and deserves a future study. The accumulated lattice variations between Mg-doped and un-doped layers can also increase the ω-scan FWHM in a layered sample. In this regard, a layered sample with a larger layer-period is expected to have a larger ω-scan FWHM, as confirmed by the data in rows 2 and 4 of Table 1. It is noted that we cannot differentiate an Mg-doped layer from an un-doped layer in the TEM images of Figure 6a–c because the difference between their lattice structures is small. Moreover, as the hillock height is only several nm, which is significantly smaller than its lateral dimension at several hundred nm, it is difficult to observe a hillock structure based on the TEM observation here.

**Figure 7:**
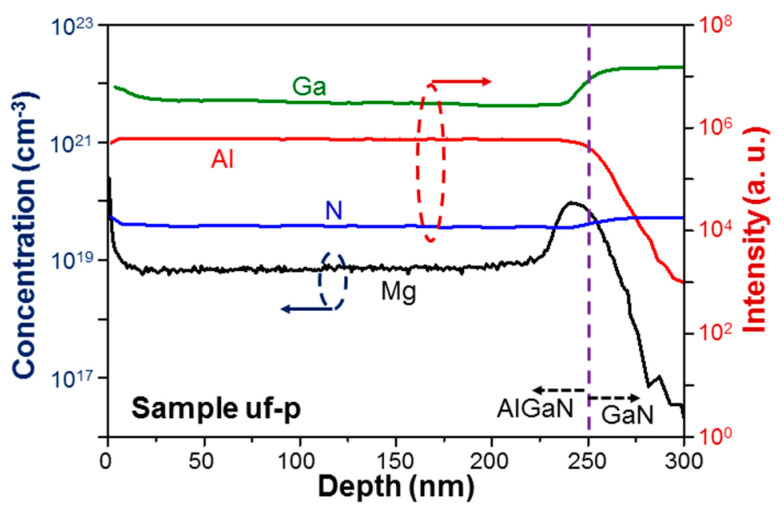
Secondary ion mass spectroscopy (SIMS) measurement results of sample uf-p with the left ordinate for the absolute Mg concentration and the right ordinate for the relative concentrations of Ga, Al, and N.

**Figure 8:**
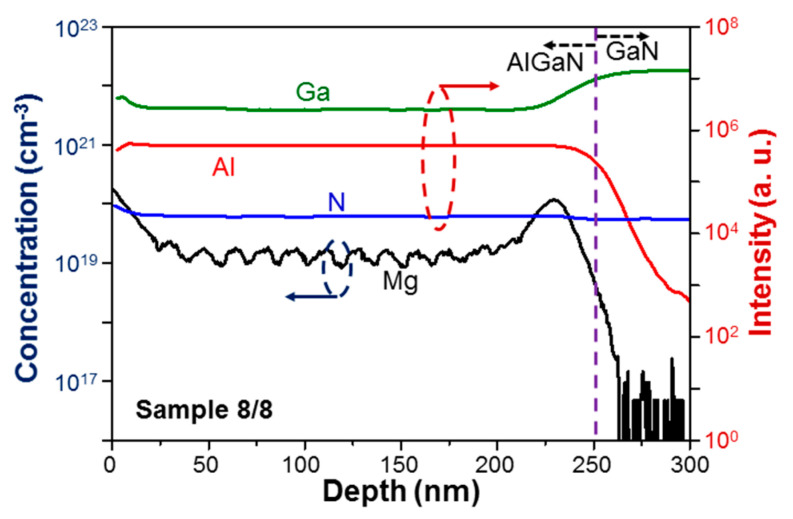
SIMS measurement results of sample 8/8 with the left ordinate for the absolute Mg concentration and the right ordinate for the relative concentrations of Ga, Al, and N.

Figure 7 and Figure 8 shows the measurement results of the secondary ion mass spectroscopy (SIMS) for sample uf-p (8/8). The SIMS measurements were performed by the company of MA-tek, Hsinchu, Taiwan. In the measurements, a data point was taken every 0.66 nm in sample depth. However, the ion diffusion within a certain depth range existed. The depth resolution was estimated to be a couple nm. In Figure 7, with the left ordinate, we can read the depth-dependent distribution of absolute Mg doping concentration in sample uf-p. With the right ordinate, we show the distributions of relative concentrations of Ga, Al, and N based on the same measurement. From the distributions of Ga and Al, we can see that the AlGaN/GaN interface is located at ~250 nm in depth, which is indicated by the vertical dashed line. The higher signal level near the sample surface in the depth range of 0–15 nm is caused by system operation and does not represent a real Mg-doping result. The higher Mg concentration around the AlGaN/GaN interface in the depth range of 230–270 nm is caused by the outburst of Mg atoms when its effusion cell shutter is opened after a long shut-down duration. The outburst of Mg atoms leads to a higher doping level for a certain duration or AlGaN thickness. Doped Mg atoms in AlGaN can diffuse into the neighboring GaN layer to produce the Mg distribution tail in the GaN template. In Figure 8 for sample 8/8, from the relative concentration distributions of Ga and Al, we can see that the AlGaN/GaN interface is also around 250 nm in depth. However, the distribution of Mg doping concentration starts at a smaller depth. This result has two attributions. First, in a layer-structured sample, we grow an un-doped AlGaN layer first. Second, because of the stronger Al-N bonding, the diffusion of Mg atoms in an un-doped AlGaN layer can be less effective, when compared with that in GaN, leading to a smaller Mg diffusion depth in GaN. The most important feature in the Mg distribution of sample 8/8 is the periodic variation of Mg concentration along depth. The variation period is indeed around 16 nm, confirming the growth of the Mg-doped/un-doped AlGaN alternating-layer structure with each layer thickness at 8 nm in this sample. The observed small oscillation range of Mg concentration has two causes. First, the depth resolution of SIMS measurement is not high enough for well resolving the Mg concentration difference between the neighboring Mg-doped and un-doped layers. Second, the residual Mg atoms in the growth chamber from the growth of an Mg-doped layer can dope into the designated un-doped AlGaN layer in the subsequent growth. Also, certain Mg atoms may diffuse from an Mg-doped layer into the neighboring un-doped layers. In other words, practically we may form a high-Mg-doped/low-Mg-doped layer structure in such a sample. Nevertheless, such a sample structure can still provide us with the advantage of high hole concentration in a high-Mg-doped layer and high hole mobility in a low-Mg-doped layer. By comparing Figure 7 and Figure 8, we can see that the Mg doping concentration in the designated Mg-doped layers of sample 8/8 is higher than that of sample uf-p. In the depth range of 15–225 nm in Figure 7, the Mg doping concentration is essentially uniform at ~9 × 10^18^ cm^−3^. In the depth range of 30–200 nm in Figure 8, the average Mg doping concentration is around 1.5 × 10^19^ cm^−3^. The depth-dependent distributions of Mg concentration in Figure 7 and Figure 8 are individually integrated to give the total doped Mg atoms per unit area at 3.67 × 10^14^ atoms/cm^2^ in sample uf-p and at 5.98 × 10^14^ atoms/cm^2^ in sample 8/8. The total doped Mg atoms in the layered structure of sample 8/8 is indeed larger than that in the uniformly-doped sample, i.e., uf-p. This result can be attributed to the aforementioned Mg outburst behavior in growing a layer-structured sample.

## 3. Hall Measurement Results

**Figure 9:**
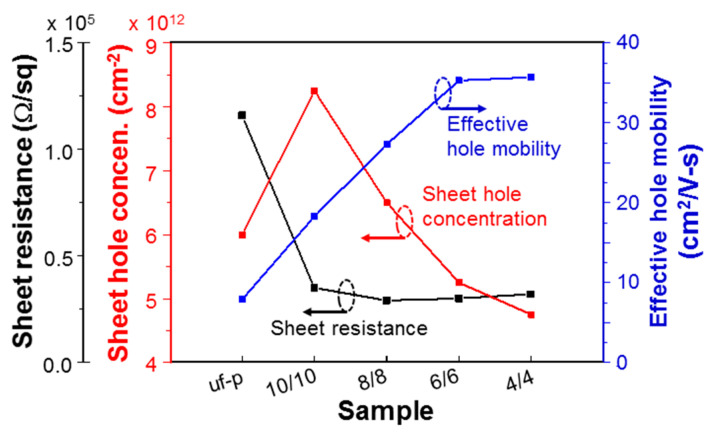
Variations of sheet resistance (with the first left ordinate), sheet hole concentration (with the second left ordinate), and effective hole mobility (with the right ordinate) of samples uf-p, 10/10, 8/8, 6/6, and 4/4.

**Figure 10:**
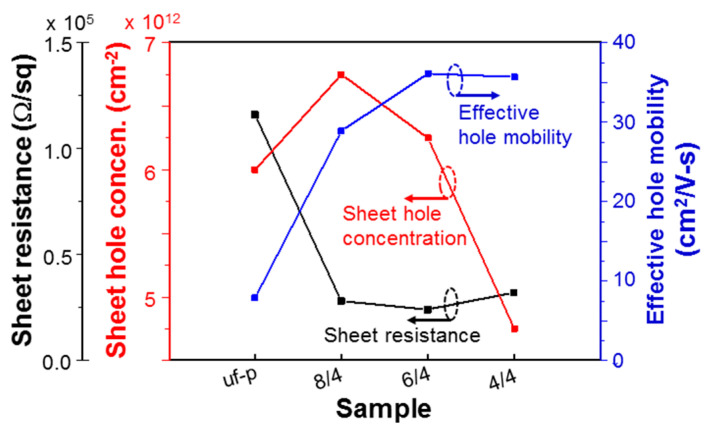
Variations of sheet resistance (with the first left ordinate), sheet hole concentration (with the second left ordinate), and effective hole mobility (with the right ordinate) of samples uf-p, 8/4, 6/4, and 4/4.

From Hall measurements, we can identify the p-type behaviors of the samples under study except sample uf-u. Also, we can obtain the sheet hole concentrations, sheet resistances, and hence effective hole mobility levels of those samples, as shown in rows 7–9 of Table 1, respectively. To clearly see the variation trends, in Figure 9, we compare the data of samples uf-p, 10/10, 8/8, 6/6, and 4/4. Moreover, in Figure 10, we compare the data of samples uf-p, 8/4, 6/4, and 4/4. In samples 10/10, 8/8, 6/6, and 4/4, the total Mg-doped layer thickness is about the same as the total un-doped layer thickness. As shown in Figure 9, the sheet hole concentration of sample 10/10 is significantly higher than that of sample uf-p. This result implies that while the Mg supply duration in growing sample 10/10 is only about one-half that of sample uf-p, the overall (sheet) hole concentration is higher in sample 10/10. This observation is also true for sample 8/8. As shown in the Mg concentration distribution around the AlGaN/GaN interface of Figure 7 or Figure 8, the Mg doping concentration reaches its maximum a certain period of time after the Mg cell shutter is opened. Although it is difficult to estimate this period of time, it can be longer than 5 min. In other words, the maximum Mg doping concentration is reached after the AlGaN growth thickness is larger than 10 nm after the Mg cell shutter is opened. In this situation, when the Mg-doped layer in a layer-structured sample is thinner than 10 nm, the cumulative Mg concentration in such a layer becomes lower. Therefore, the sheet hole concentration for a layer-structured sample shown in Figure 9 decreases with decreasing Mg-doped layer thickness. This variation trend can also be observed in Figure 10. The decreasing fraction of total Mg-doped layer thickness from sample 8/4 to 4/4 further reduces the sheet hole concentration. The discussions above are based on the reasonable assumption that the hole generation efficiency is fixed among all the Mg-doped samples under study. It is noted that a two-dimensional electron gas (2DEG) exists between the overgrown AlGaN structure and the GaN template. However, the effect of this 2DEG on the Hall measurement result is expected to be small because of the formation of a depletion region between the overgrown p-type AlGaN and the 2DEG. The depletion region, which lies on the AlGaN side, minimizes the effect of the 2DEG on a Hall measurement. Meanwhile, in a grown layered structure, the injected current density decays along AlGaN depth in a Hall measurement. Therefore, in the current study, the effects of the 2DEG on the Hall measurement results must be small.

In row 8 of Table 1 and Figure 9 and Figure 10, we can see that the sheet resistance levels of all the layer-structured samples are significantly lower than that of sample uf-p. The lowest sheet resistance of 0.24 × 10^5^ Ω/square is obtained in sample 6/4, which is 4.83 times lower than that of 1.16 × 10^5^ Ω/square in sample uf-p. It is difficult to identify a clear variation trend for the sheet resistance data among those layer-structured samples. In row 9 of Table 1 and Figure 9 and Figure 10, we also show the variation of effective hole mobility, which is obtained through Equation (1). In Figure 9 and Figure 10, one can see that except sample 4/4, the effective hole mobility increases with decreasing layer thickness. The effective hole mobility of sample 4/4 is close to those of samples 6/4 and 6/6. The highest effective hole mobility of 36.1 cm^2^/V-s is obtained in sample 6/4, which is 4.57 times that of 7.9 cm^2^/V-s in sample uf-p. Since the higher effective hole mobility in a layer-structured sample is caused by the current flows in the un-doped AlGaN layers, its level is controlled by the overall thickness fraction of un-doped layers and the fraction of holes diffused into the un-doped layers from the Mg-doped layers. Between samples 8/4 and 6/4, the larger thickness fraction of un-doped layer in sample 6/4 leads to a higher effective mobility level. Among samples 10/10, 8/8, and 6/6, the increasing effective hole mobility with decreasing layer thickness can be attributed to the increasing fraction of holes diffused into un-doped layers. The fraction of hole diffusion saturates when the layer thickness decreases from 6 to 4 nm such that the mobility levels of samples 4/4, 6/6, and 6/4 are about the same.

The sheet resistance in a layer-structured sample can be quite low if both sheet hole concentration and effective hole mobility are high. However, as shown in Table 1, among the layer-structured samples, the highest sheet hole concentration in sample 10/10 corresponds to the lowest effective hole mobility, leading to the highest sheet resistance. Although the sheet hole concentration of sample 6/4 is not the highest, its highest effective hole mobility results in the lowest sheet resistance. In the bottom two rows of Table 1, we show the effective hole concentration and effective resistivity of those p-type samples by assuming that the hole concentration and mobility are uniform in the whole AlGaN layer of each sample. This issue will be further discussed in the next section. The effective resistivity of sample 6/4 can be reduced to 0.61 Ω-cm.

## 4. Discussions

In Table 2, we compare the results of effective hole concentration, effective resistivity, and effective hole mobility between the GaN and AlGaN (~25% Al) samples of the similar layer structures (samples 8/8 and 4/4). The results of the GaN samples are quoted from ref. [28]. It is noted that the MBE growth conditions for the GaN and AlGaN samples are quite different. Therefore, our comparison between the GaN and AlGaN samples focuses at the effects of the layer structure. In Table 2, the numbers inside the parentheses show the ratios of the results of layer-structured samples with respect to the corresponding values of the uniformly-doped sample in either GaN or AlGaN sample group. From the results of effective hole concentration in Table 2, one can see that the enhancement ratio of hole concentration in a layer-structured AlGaN sample due to Mg outburst is smaller, when compared with the corresponding GaN sample. In particular, the effective hole concentration in the AlGaN 4/4 sample is lower than that of the corresponding uniformly-doped AlGaN sample. This result can be attributed to the smaller Mg doping amount in AlGaN within a short Mg-shutter open duration, when compared to GaN. It is noted that the Ga-N bonding energy of 2.2 eV is smaller than that of the Al-N bonding energy of 2.88 eV [16,30,31]. Therefore, in an Mg doping process, it is relatively more difficult to replace an Al atom by an Mg atom, when compared with the replacement of a Ga atom. In other words, the doping efficiency in AlGaN can be lower than that in GaN, particularly within a short doping duration. From the results of effective resistivity and hole mobility in Table 2, one can see that the effective resistivity can be more significantly reduced through the formation of a layer structure in GaN, when compared with AlGaN. In GaN, a thinner layer leads to a low resistivity level and a higher effective hole mobility. However, in AlGaN, the variation trend is not straightforward. In AlGaN 8/8 sample, the effective resistivity is reduced and the effective hole mobility is increased, when compared with the corresponding uniformly-doped sample. However, in AlGaN 4/4 sample, although the effective hole mobility is further increased, the resistivity is not further decreased. Besides the aforementioned lower doping efficiency, this result can be attributed to the relatively poorer crystal quality in this sample. The high V-pit density and large ω-scan FWHM in sample 4/4, as shown in Figure 4d2 and Table 1, imply the high defect density in this sample. Defects in Mg-doped GaN or AlGaN can compensate generated holes and hence degrade p-type performance.

In the bottom row of Table 1, we show the results of effective resistivity of all those p-type samples. These results are obtained by assuming that the depth distributions of hole concentration and mobility are uniform in the whole AlGaN layer of a sample. However, in a layer-structured sample, the hole concentrations or mobility levels are different between an Mg-doped and an un-doped AlGaN layers. In particular, the lower hole concentration in an un-doped layer may result in resistance to vertical current flow in a Hall measurement. Therefore, the lateral-flow current density is expected to decrease with sample depth. In other words, the effective depth of current distribution in a layer-structured sample is smaller than the total AlGaN thickness. Therefore, if we define the p-type layer thickness in a layer-structured sample as its effective current penetration depth, the effective resistivity of this sample becomes lower than what is shown in the bottom row of Table 1. In an earlier study on layer-structured p-type GaN, based on this definition of p-type layer thickness, a model was proposed for evaluating the current penetration depth and the lower effective resistivity of a layer-structured sample [32]. However, it is noted that in a Hall measurement, what we can directly measure are sheet carrier concentration and sheet resistance. The determinations of effective carrier concentration and effective resistivity rely on the definition of p-type layer thickness (either the whole layered structure or the effective current penetration depth). The comparisons between the two different carrier concentration and resistivity results based on the different definitions of p-type layer thickness can provide us with certain insights about the conduction behavior of an Mg-doped/un-doped layer structure, from which we can better design such a layered structure, including the layer thickness and layer period number. Nevertheless, in practical application, only the sheet resistance is of great concern. The determinations of effective carrier concentration and effective resistivity are not very crucial in the effort of improving the p-type behavior.

It is noted that Hall measurement is typically used for evaluating the resistivity of a p- or n-type layer. In the scheme of such a measurement, mainly the lateral current flow behavior is monitored. In device application, a bias voltage is applied across the layers of an alternating-layer structure under study. In this situation, the vertical conductivity relies on the conductivity in an un-doped layer, which depends on the hole diffusion range from an Mg-doped layer into the neighboring un-doped layers [32]. In other words, the thickness of an un-doped layer should be small enough, such as in the range of 2–4 nm. Under this condition, the vertical resistivity can be in the same order of magnitude as that in the lateral direction.

**Figure 11:**
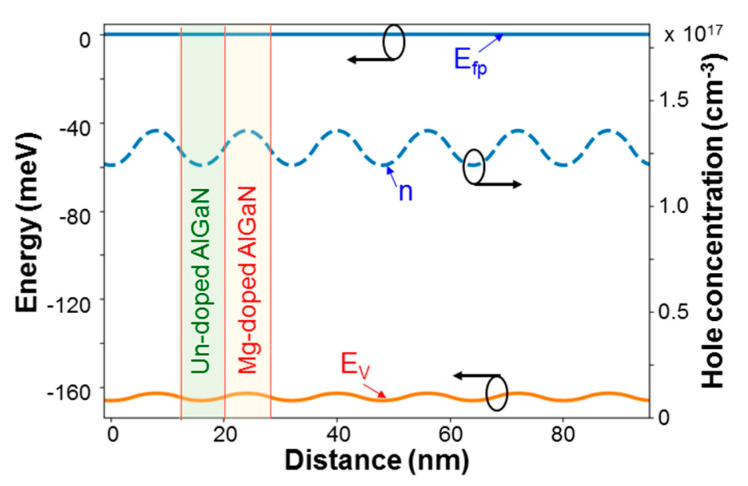
Simulation results of valence band energy and hole concentration distributions in sample 8/8 by assuming that the Mg doping concentration in an Mg-doped layer is 2 × 10^19^ cm^−3^ and the acceptor activation energy in AlGaN of 25% in Al content is 240 meV.

Figure 11 shows the simulation results of *E_fp_*, *n*, and *E_V_* in sample 8/8 by assuming that the Mg doping concentration in an Mg-doped layer is 2 × 10^19^ cm^−3^ and the acceptor activation energy in AlGaN of 25% in Al content is 240 meV [33]. Here, *E_fp_* represents the Fermi level energy of hole, *n* stands for the hole concentration, and *E_V_* denotes the valence band energy. The oscillating behavior of hole concentration in Figure 11 indicates the diffusion of holes from an Mg-doped layer into the neighboring un-doped layers. From the curve of *E_V_*, we can see that the valence band energy at the center of an Mg doped layer is lower than that at the center of an un-doped layer by 3.326 meV. This energy barrier is small such that holes generated in an Mg-doped layer can easily diffuse into the neighboring un-doped layers at room temperature.

## 5. Conclusions

In summary, seven p-type AlGaN samples of ~25% in Al content, including six samples with Mg-doped/un-doped AlGaN alternating-layer structures of different layer thicknesses, have been fabricated based on MBE growth for comparing their p-type performances. The sheet resistance was reduced and the effective hole mobility was increased in a layer-structured sample, when compared with the reference sample of uniform Mg doping. The improved p-type performance in a layer-structured sample was caused by the diffusion of holes generated in an Mg-doped layer into the neighboring un-doped layers, in which holes are able to transport with significantly higher hole mobility. Among the layer-structured samples of different layer-thickness combinations, the sample of 6/4 nm in Mg-doped/un-doped thicknesses led to the lowest sheet resistance (the highest effective hole mobility), which was 4.83 times lower (4.57 times higher) when compared with the sample of uniform doping.

## Figures and Tables

**Table 1:** Structures and characterization results of the samples under study.

Sample	uf-u	uf-p	10/10	8/8	6/6	4/4	8/4	6/4
Period number	---	---	12	15	20	30	20	24
AlGaN thickness (nm)	250	250	250	252	254	256	252	254
XRD ω-scan FWHM (arcsec)	256.4	263.0	278.4	295.4	298.5	308.8	298.0	302.2
Roughness (nm)	1.3/0.9	1.1/0.8	2.0/2.5	1.7/1.5	1.8/1.7	1.6/1.3	1.8/2.1	2.0/1.5
V-pit (hillock) density (10^8^ cm^−2^)	(1.33)	(2.22)	3.7	1.1	2.0	9.5	3.4	3.2
Sheet hole concentration (×10^12^ cm^−2^)	---	6.0	8.25	6.5	5.25	4.75	6.75	6.25
Sheet resistance (×10^5^ Ω/sq)	---	1.16	0.35	0.29	0.30	0.32	0.28	0.24
Effective hole mobility (cm^2^/V-s)	---	7.9	18.3	27.3	35.3	35.7	28.9	36.1
Effective hole concentration (×10^17^ cm^−3^)	---	2.4	3.3	2.6	2.1	1.9	2.7	2.5
Effective resistivity (Ω-cm)	---	2.9	0.88	0.73	0.74	0.79	0.70	0.61

**Table 2:** Comparison of the results of effective hole concentration, effective resistivity, and effective hole mobility of samples uf-p, 8/8, and 4/4 between GaN and AlGaN.

Sample	GaN			AlGaN		
	uf-p	8/8	4/4	uf-p	8/8	4/4
Effective hole concentration (×10^17^ cm^−3^)	2.84 (1)	9.17 (3.23)	7.39 (2.60)	2.4 (1)	2.6 (1.08)	1.9 (0.79)
Effective resistivity (Ω-cm)	1.55 (1)	0.191 (0.123)	0.063 (0.041)	2.9 (1)	0.73 (0.252)	0.79 (0.272)
Effective hole mobility (cm^2^/V-s)	14.2 (1)	35.5 (2.50)	135.3 (9.53)	7.9 (1)	27.3 (3.46)	35.7 (4.52)

## Data Availability

All the data supporting reported results can be found in the text of this paper.
